# Scalable high-throughput identification of genetic targets by network filtering

**DOI:** 10.1186/1471-2105-14-S8-S5

**Published:** 2013-05-09

**Authors:** Vitoantonio Bevilacqua, Paolo Pannarale

**Affiliations:** 1 Department of Electrical and Electronics, Polytechnic of Bari, Via E. Orabona, 4, 70125 Bari, Italy

## Abstract

**Background:**

Discovering the molecular targets of compounds or the cause of physiological conditions, among the multitude of known genes, is one of the major challenges of bioinformatics. One of the most common approaches to this problem is finding sets of differentially expressed, and more recently differentially co-expressed, genes. Other approaches require libraries of genetic mutants or require to perform a large number of assays. Another elegant approach is the filtering of mRNA expression profiles using reverse-engineered gene network models of the target cell. This approach has the advantage of not needing control samples, libraries or numerous assays. Nevertheless, the impementations of this strategy proposed so far are computationally demanding. Moreover the user has to arbitrarily choose a threshold on the number of potentially relevant genes from the algorithm output.

**Results:**

Our solution, while performing comparably to state of the art algorithms in terms of discovered targets, is more efficient in terms of memory and time consumption. The proposed algorithm computes the likelihood associated to each gene and outputs to the user only the list of likely perturbed genes.

**Conclusions:**

The proposed algorithm is a valid alternative to existing algorithms and is particularly suited to contemporary gene expression microarrays, given the number of probe sets in each chip, also when executed on common desktop computers.

## Background

The identification of compound mode of action is crucial in the development of a new drug. It allows increasing affinity with desired targets and reduces side effects. On the other hand the study of complex diseases etiology may benefit from high-throughput screenings of genomic profiles [1], for example for the identification of proto-oncogenes [2].

The mode of action of a drug or the cause of diseases involving the genetic machinery can be seen as the deviation or perturbation of the genetic behavior of the cell, from a reference one, towards a new state. Differential expression or co-expression [3,4] is based on a comparison of gene expression levels before and after this state transition. However, whole-genome expression profiles do not distinguish the genes targeted by a compound from the indirectly regulated genes. The latters may often present differences of expression even bigger in magnitude than the direct targets. For this reason a common step following the obtainment of an expression profile is its comparison with a panel of profiles with known mode of action or genetic mutations. These association analysis techniques are demonstrated to be very effective [5], but they need a large panel of profiles to compare with, and they are not helpful when the MoA (Mode of Action of a compound, drug, etc.) was previously unreported. This method has been extended in order to account for multiple sources of data [6,7].

Other techniques, requiring a large amount of assays include haploinsufficiency profiling [8] and chemical-genetic interaction mapping [9].

Finally, other techniques use a reverse engineered model of regulatory interactions to analyze the expression profile of perturbed cells, or to create new drugs that target, for example, a pathogen organism, while minimizing the damage to the host [10]. Regulatory models have been proved to be a valuable source of information not only in drug discovery, but also in cancer classification [11] or clustering [12] and many other bioinformatics applications.

The algorithms that identify the MoA of existing molecules, using quantitative gene network models, assume that training profiles are obtained in steady state following a variety of treatments, including compounds, RNAi, and gene-specific mutations. The quantitative gene network reverse engineering algorithms can be divided in two categories [13]: those requiring knowledge of the gene targeted in each training experiment and those, like MNI [14] and Ssem-lasso [15], which improve flexibility, not requiring this additional information. This improved flexibility enables their application to higher model organisms, where gene-specific perturbations are more difficult to implement.

To infer a network model without requiring gene-specific perturbations, the MNI algorithm employs an iterative procedure analogous to the Expectation Maximization (EM) algorithm: it first predicts the targets of the treatment using an assumed network model, and then uses those predicted targets to estimate a better model. The interaction model is reconstructed by SVD [16] factorization. The procedure repeats until convergence criteria are met. This approach requires a non-trivial amount of expert supervision to tune it appropriately.

The second option, SSEM-LASSO, use a formal statistical modeling framework and an associated inferential strategy for the problem of predicting directly perturbed genes from DNA microarray expression profiles. The SSEM algorithm is considered particularly well suited by the authors for the given application, given the peculiarity of gene regulation: simultaneity, because each gene may act as both dependent and independent variable, and sparseness, given the low numbers of regulators per gene and the low expected number of directly targeted genes for a typical perturbation. Thus sparse simultaneous equation models (SSEMs).

For both approaches, once the regulatory model is trained, the expression profile of a test compound is filtered, in essence, checking the expression level of each gene in the cell (relative to the level of all other genes in the cell) for consistency with regulatory influences embodied in the trained regulatory model. The genes are then ranked by a measure of their level of consistency with the expected behavior, based on the model. The inconsistency is attributed to the external influence of the compound on those genes.

Our mode of action identification procedure is based on machine learning algorithms. The samples are divided in training and testing samples. Within the training samples, a sub list of potentially connected genes is selected through classical feature selection algorithms. A Support Vector Machine Regression [17] is performed with the short list of regulators as features. In the validation step the regression model is evaluated on the remaining samples. The validation residuals are computed as the difference of the predicted value from the actual one. The residuals are used to compute the residual distribution function for each gene. The distribution function is used to compute the likelihood of the residual for each gene of the treated sample. The genes whose residuals have a low likelihood are returned to the user. These genes are further examined in order to found deregulated pathways.

The method has been tested on simulated datasets and on the combination of two publicly available, two-color cDNA whole-genome yeast expression data sets: a compendium of 300 profiles of gene deletions, titratable promoter insertions and drug compound treatments from Hughes et al. [5] and a second set of 215 titratable promoter insertions in essential genes from Mnaimneh et al. [18]. Finally the algorithm has been tested on an Affymetrix yeast S. cerevisiae compendium of 904 samples.

We compared the performance of MNI, SSEM-LASSO and our algorithm, by testing its ability to predict the gene targets of the 11 promoter insertions from the Hughes compendium. We next applied the algorithms to identify probable targets of drug compounds.

The algorithms are compared also in terms of performances both in the time and the space domain. These comparisons have been performed for various combinations on the number of genes and experiments using simulated datasets.

The algorithm showed sensitivity and specificity comparable to the other methods. On the other hand CPU and memory consumption of our approach make it a good choice given the reasonable trade-off between time and space demands.

## Results and discussion

### Simulation results

**Figure 1:**
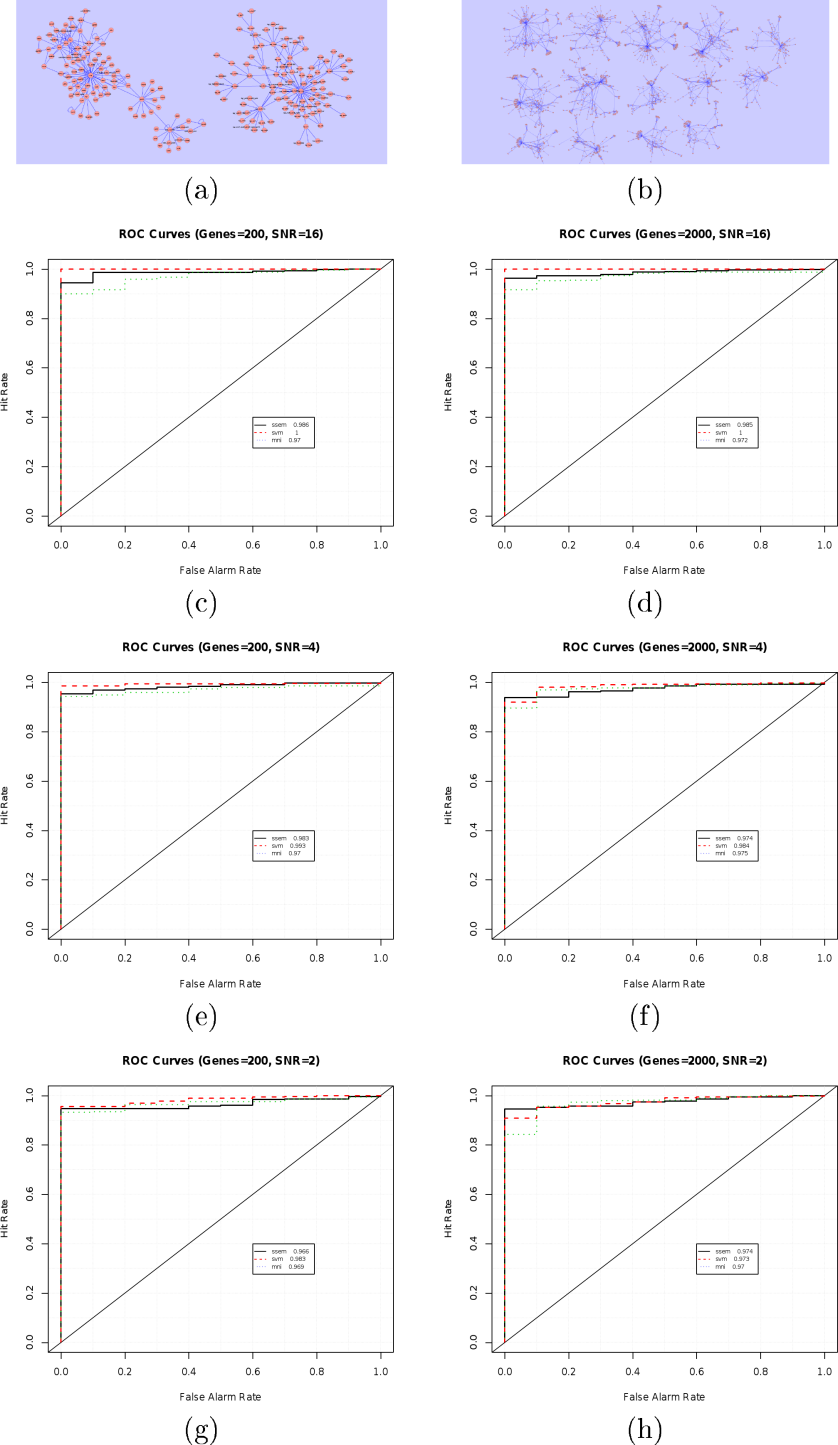
**Sensitivity and specificity for simulated datasets**. Figures c, e and g show the ROC curve for the 200 genes network (a) for respectively 16, 4 and 2 SNR. Figures d, f and h show the ROC curve for the 2000 genes network (b) for respectively 16, 4 and 2 SNR. The ROC lines are respectively continuous, dashed and dotted for SSEM, SVM and MNI.

A lognormal noise with expected value equal to 10% of the raw expression value has been added to the simulated datasets. In each experiment one gene has been randomly selected and its value has been modified by adding or subtracting a quantity proportional, with a given ratio to the experimental noise. In this way we obtained datasets with different values of Signal to Noise Ratio. The SNR has been set to 16, 4 and 2. The three datasets have been generated for two synthetic networks, the first with 200 genes and 321 interactions and the second having 2000 genes and 3082 interactions. The generated networks are reported in Figure 1a and 1b respectively. The ROC curves for the 200 gene network datasets are reported on the left and those for the 2000 genes network on the right. The plots are ordered by decreasing SNR. In the first row, the datasets with SNR = 16 have given a perfect 100% AUC. In fact the p-values for the perturbed genes were extremely low. The AUC decreased to 99% and 98% for SNR equals to 4 and 2 of the 200 genes network (Figure 1 e,g). The AUCs for the large gene network were 98% and 97% for SNR of 4 and 2 (Figure 1 f,h). The results have been obtained generating multiple datasets, each one made up of 99 unperturbed and 1 perturbed experiments. The perturbation is an inconsistency of the expression values with the underlying gene network. In the training phase only the unperturbed experiments were used for building the interaction model.

The sensitivity/specificity has been calculated using the *verification *package of the statistical software R. The values of our algorithm are comparable to those of the competing methods, as shown in Figure 1. A slight improvement is due, in our opinion, to the residual estimation strategy that can control the false positive rate and on the non-linearity of the modeling strategy which can better represent complex interaction dynamics. The ROC curves for 16 SNR are very accurate, such ratio is very high given that the noise is about 10% of the signal; it means that the perturbation is 1.6 times the original value. Unfortunately the improvements over the competing methods for the synthetic datasets are not followed by comparable improvements for the real datasets, as showed in the following sections.

### Microarray compendia results

In analyzing the *microarray compendia *predictions for compound treatments, we considered as targets both the pathways that are significantly overrepresented among the perturbed genes and the genes themselves. Pathways are identified as significantly overrepresented Gene Ontology processes among the highly ranked genes.

The results for the promoter insertions are reported in table 1. The SVM algorithm identified all the target genes with an efficacy comparable to those of the competing methods. Although the mean rank was 3.2 and the mean rank of the next best performing method, MNI, was 5.5, we can't state that the SVM method performed better, given that a two ample t-test of the ranks couldn't reject the null hypothesis. On the other hand the number of results for SVM is lower than for MNI and SSEM-LASSO with p*<*0.05.

**Table 1:** Results for genetic perturbations

Promoter mutant	Target	MNI		ssem-lasso		SVM		
		
		Rank	Results	Rank	Results	Rank	Results	N°attributes
tet-CMD1	CMD1	1	100	>MNI	100	2	100	29
tet-AUR1	AUR1	1	100	>MNI	100	1	100	40
tet-CDC42	CDC42	1	100	>MNI	100	1	100	26
tet-ERG11	ERG11	42	100	>MNI	100	10	100	15
tet-FKS1	FKS1	1	100	>MNI	100	2	17	21
tet-HMG2	HMG2	1	100	>MNI	100	10	100	34
tet-IDI1	IDI1	1	100	>MNI	100	1	16	28
tet-KAR2	KAR2	1	100	>MNI	100	1	100	29
tet-PMA1	PMA1	6	100	>MNI	100	5	100	23
tet-RHO1	RHO1	4	100	>MNI	100	1	10	32
tet-YEF3	YEF3	1	100	>MNI	100	1	36	44

Our algorithm has also been tested on real compound treated experiments. The compounds used with cDNA arrays were lovastatin, terbinafine, itraconazole, hydroxyurea and tunicamycin.

Lovastatin is structurally similar to the HMG, a substituent of the endogenous substrate of HMG-CoA reductase. Lovastatin is a prodrug that is activated in vivo via hydrolysis of the lactone ring. The hydrolyzed lactone ring mimics the tetrahedral intermediate produced by the reductase allowing the agent to bind with 20,000 times greater affinity than its natural substrate. The bicyclic portion of lovastatin binds to the coenzyme A portion of the active site.

Terbinafine is hypothesized to act by inhibiting squalene monooxygenase, thus blocking the biosynthesis of ergosterol, an essential component of fungal cell membranes. This inhibition also results in an accumulation of squalene, which is a substrate catalyzed to 2,3-oxydo squalene by squalene monooxygenase. The resultant high concentration of squalene and decreased amount of ergosterol are both thought to contribute to terbinafine's antifungal activity.

Itraconazole interacts with 14-*α *demethylase, a cytochrome P-450 enzyme necessary to convert lanosterol to ergosterol. As ergosterol is an essential component of the fungal cell membrane, inhibition of its synthesis results in increased cellular permeability causing leakage of cellular contents. Itraconazole may also inhibit endogenous respiration, interact with membrane phospholipids, inhibit the transformation of yeasts to mycelial forms, inhibit purine uptake, and impair triglyceride and/or phospholipid biosynthesis.

Hydroxyurea is converted to a free radical nitroxide (NO) in vivo, and transported by diffusion into cells where it quenches the tyrosyl free radical at the active site of the M2 protein subunit of ribonucleotide reductase, inactivating the enzyme. The entire replicase complex, including ribonucleotide reductase, is inactivated and DNA synthesis is selectively inhibited, producing cell death in S phase and synchronization of the fraction of cells that survive. Repair of DNA damaged by chemicals or irradiation is also inhibited by hydroxyurea, offering potential synergy between hydroxyurea and radiation or alkylating agents. Hydroxyurea also increases the level of fetal hemoglobin, leading to a reduction in the incidence of vasoocclusive crises in sickle cell anemia. Levels of fetal hemoglobin increase in response to activation of soluble guanylyl cyclase (sGC) by hydroxyurea-derived NO.

Tunicamycin is an inhibitor of bacterial and eukaryote N-acetylglucosamine transferases; preventing formation of N-acetylglucosamine lipid intermediates and glycosylation of newly synthesized glycoproteins. Tunicamycin blocks the formation of protein N-glycosidic linkages by inhibiting the transfer of N-acetylglycosamine 1-phosphate to dilichol monophosphate.

Unlike promoter insertions, which directly influence transcription, compounds predominantly affect protein activity and only indirectly influence transcription. As a result, the algorithm is more likely to identify genes in the same pathway as the affected protein rather than the target itself, such as transcriptionally regulated genes downstream of the target protein. On the other hand, when transcriptional feedback regulation is present in the pathway containing the targeted gene, it is likely that the algorithm will also assign a high rank to the targeted gene product [15].

For the drug treated samples our algorithm identified the target genes for only 3/7 of the compound targets, while the competing methods performed better with 5/7 of correctly identified genes. The results are showed in table 2.

**Table 2:** Results for drug perturbations of cDNA arrays

Drug	Target	MNI		Ssem-lasso		SVM		
		
		Rank	Results	Rank	Results	Rank	Results	N°attributes
Terbinafine	ERG1	5	100	-	100	-	57	20
Lovastatin	HMG2	30	100	31	100	-	59	34
Lovastatin	HMG1	-	100	89	100	-	59	33
Itraconazole	ERG11	2	100	17	100	27	100	15
Hydroxyurea	RNR2	2	100	20	100	26	83	22
Hydroxyurea	RNR4	6	100	4	100	7	83	30
Tunicamycin	ALG7	-	100	-	100	-	59	10

Even if the performance of mode of action identification of our algorithm were worse than those of MNI or SSEM-LASSO in the identification of targeted genes, surprisingly it was more effective in the identification of the involved pathways (table 3). In parentheses the number of genes associated to each GO Term is reported. For the identified pathways an average ratio of GO Term dimension less than 1/3 was obtained with respect to the pathways identified by MNI. Our algorithm failed the identification of the pathway targeted by lovastatin. On the contrary MNI identifies a pathway, but it is very generic.

**Table 3:** Pathway analysis of cDNA arrays

Drug	SVM Pathways	MNI Pathways	Known MoA
Terbinafine	Ergosterol biosynthetic process (27)	Steroid metabolism (2130)	Inhibition of squalene monooxygenase, thus blocking the biosynthesis of ergosterol

Lovastatin	-	Lipid metabolism (16244)	Inhibition of HMG-CoA reductase

Itraconazole	Ergosterol biosynthetic process (256)	Steroid metabolism (2130)	Interaction with 14-*α *demethylase an enzyme necessary to convert lanosterol to ergosterol.

Hydroxyurea	Deoxyribonucleotide biosynthetic process (704)	Dna replication (5480)	Inhibition of ribonucleotide reductase and consequently of DNA synthesis

Tunicamycin	Cellular nitrogen compound catabolic process (6678), Protein targeting to ER (585)	Protein-ER targeting (585)	N-linked glycosylation

The numbers of selected attributes that make important contribution to the performance of the regression models of the known target genes are reported in table 1 for the promoter insertions and on table 2 for the drug treatments. The exact list is available upon request to the authors.

The Affymetrix compendium was tested by the samples treated with Caspofungin, Thiolutin, Nocodazole and Benomyl.

Caspofungin is an antifungal drug, the first of a new class termed the echinocandins. It shows activity against infections with Aspergillus and Candida, and works by inhibiting *β *(1,3)-D-Glucan synthesis of the fungal cell wall, acted by the FKS1 and GSC2 components. Thiolutin is a sulfur-containing antibiotic, which is a potent inhibitor of bacterial and yeast RNA polymerases. A known target is the RPB10 gene. Nocodazole is an anti-neoplastic agent which exerts its effect in cells by interfering with the polymerization of microtubules. Benomyl binds to microtubules, interfering with cell functions, such as meiosis and intracellular transportation. The known genetic target for nocodazole and benomyl is the TUB1 gene.

The results reported in table 4 show very poor results for the Affymetrix compendium for the three methods. The ranks for our method are reported independently from their statistical significativity. The analysis of GO terms enrichment did not lead to significative results in the case of Affymetrix chips.

**Table 4:** Results for drug perturbations of the Affymetrix compendium

Drug	Target	MNI	Ssem-lasso	SVM
		
			Ranks	
Caspofungin	FKS1	-	27	532

Caspofungin	GSC2	-	942	3249

Thiolutin	RPB10	-	1494	724

Nocodazole	TUB1	-	978	5980

Benomyl	TUB1	-	978	918

### Performance comparison

**Figure 2:**
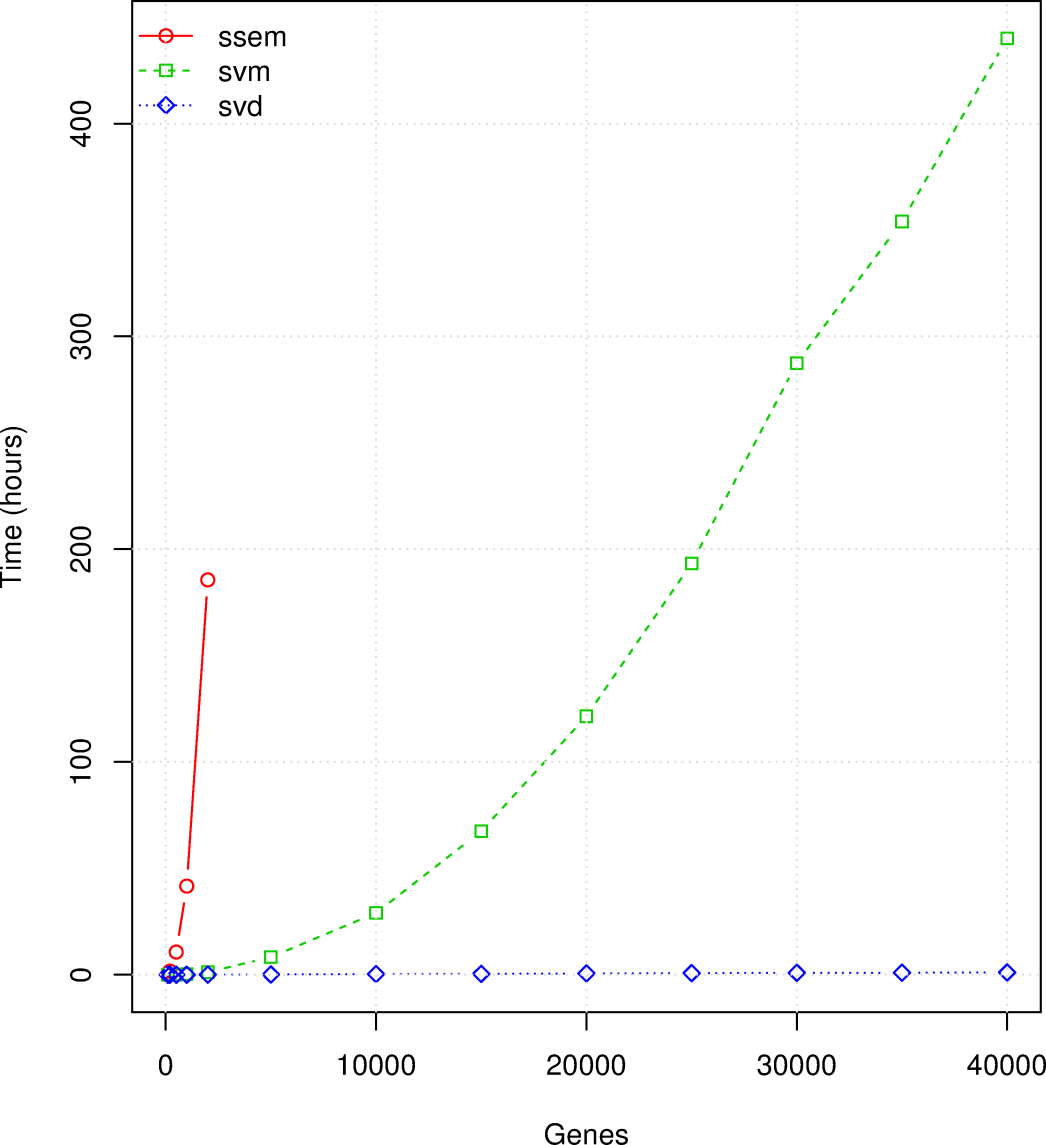
**Time performance comparison for 1500 experiments**.

**Figure 3:**
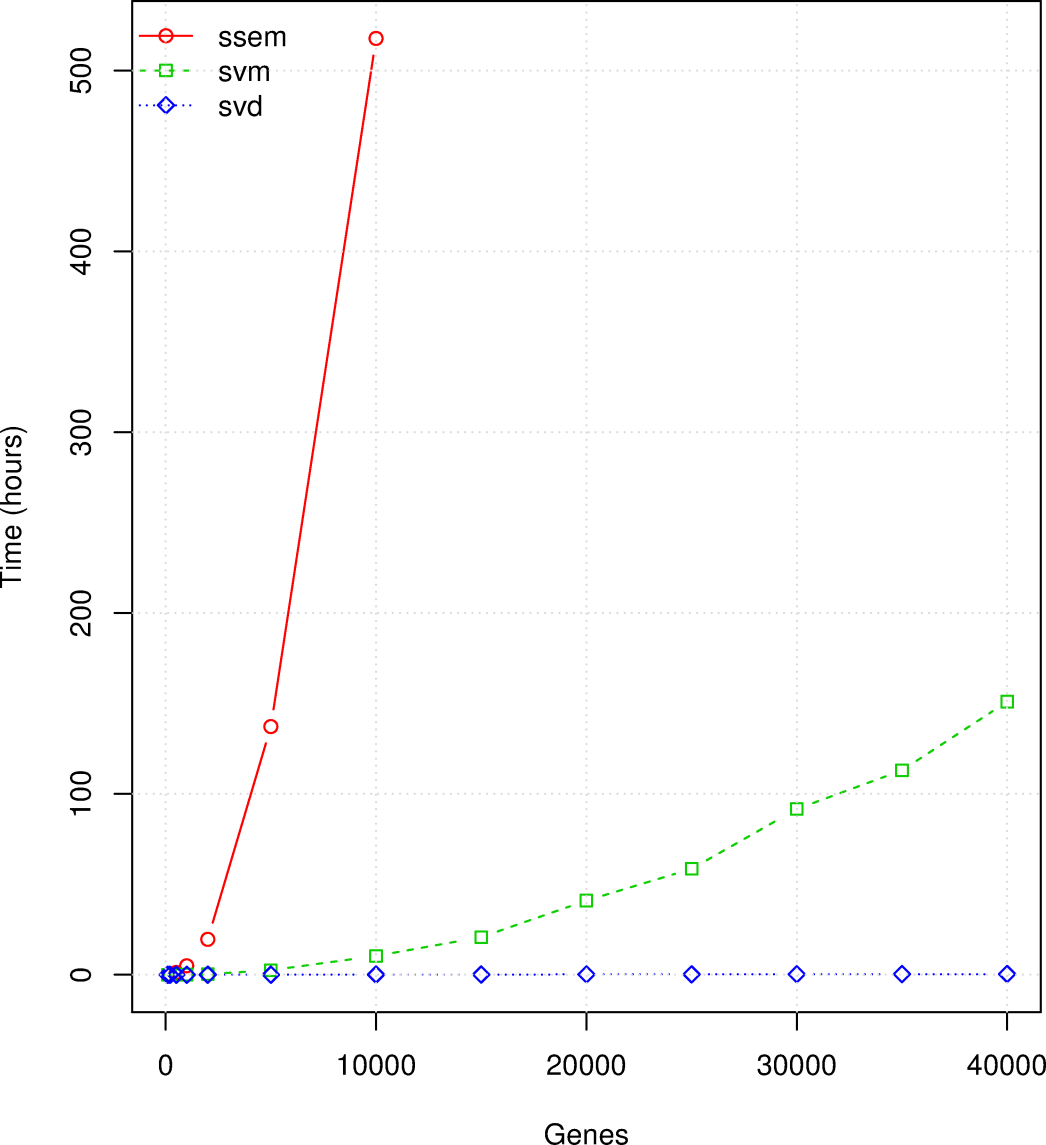
**Time performance comparison for 500 experiments**.

**Figure 4:**
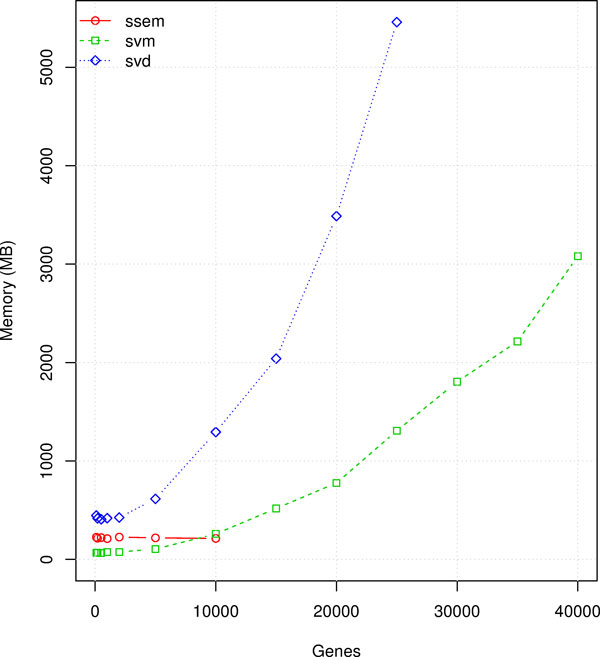
**Memory performance comparison**.

Performance results are summarized in Figures 2, 3 and 4. The "ssem" label in the figures refers to the SSEM-LASSO algorithm by [15], the "svd" label refers to the work of [14], elsewhere named MNI (MNI relies on Singular Value Decomposition for modeling the interactions, as described in the previous sections), "svm" refers to our work. The tests have been realized for a number of genes varying from few hundreds to 40 thousands and for a number of 500 and 1500 experiments.

CPU consumption (Figures 2 and 3) is affected by both the number of experiments and the number of probe sets in the chip. Independently from the dataset dimension, the best performing method is MNI. The SVM regression algorithm is markedly more demanding than MNI, but, over both, SSEM-LASSO requires several orders of magnitude more CPU. It is heavily affected by both the number of genes and the number of experiments. Indeed a Sun Grid Engine cluster of 94 dual-processor 2GB-RAM machines was employed in the original work by Cosgrove et al. [15]. Estimating the interaction model took about 4 days using 50 nodes of the cluster for a dataset of 6681 genes × 647 experiments. As a consequence we had to stop the simulations after a reasonable amount of days. The use of SSEM-LASSO by an ordinary computer is actually unfeasible and this makes its use prohibitive.

The memory test results were unaffected by the number of experiments and the results showed refer to the 500 experiments dataset (Figure 4). The tests show that MNI, while being favorable in terms of time, requires an amount of memory which rises rapidly with the number of genes in the chip. This makes the MNI execution prohibitive for a common desktop PC given that the number of probe sets on microarray chips is rapidly increasing and is well above the 40 thousands probe sets since the begin of the past decade.

Our algorithm scales well if considering both time and memory consumption. In its Java implementation we could use the multi-thread paradigm, where the number of threads for the yeast dataset execution, was limited from the number of CPU cores rather than from the available memory. Memory requirements are due to the correlation feature selection step of the gene network topology configuration, thus it is configurable on the number of genes to be retained after the first gain ratio filtering.

## Conclusions

Gene network filtering of expression profiles has been demonstrated to be a valuable tool for the identification of compound mode of action. In particular these tools can distinguish the direct target of the compounds better than simply detecting the gene expression ratio with respect to some reference sample.

MNI and SSEM-LASSO have showed good rates of target identification and anyway an improvement over the tested null methods. While SSEM-LASSO performed better than MNI on simulated datasets, it has been outperformed by the latter on real yeast two-color array datasets. Our approach demonstrated very good performances on synthetic datasets, and results comparable to MNI on promoter insertion samples. Unfortunately the SVM based algorithm obtained a lower rate of success on compound treated samples. Nevertheless when it comes to the pathway identification we showed that we identified more specific pathways than MNI on cDNA arrays. Cosgrove et al. [15] conducted a Gene Ontology term enrichment for the top 100 ranked genes for all drug perturbations with known targets, but they did not observe enrichment of the appropriate terms.

Cosgrove et al. [15] reported a major improvement over the null method on affymetrix datasets rather than two-color arrays. However while in the first case the target rank was in most cases between five hundreds and one thousand, in the second it was below one hundred. This actually suggests that gene network filtering hasn't been applied successfully on single-channel microarray data and that much work can be done in this sense.

One of the major drawbacks of MNI and SSEM-LASSO is their poor scalability both in terms of memory and elaboration time. They can't be applied to contemporary gene expression microarrays, given the number of probe sets in each chip, by means of common desktop computers. On the contrary the behavior of our strategy is acceptable also in the absence of more powerful means.

## Methods

**Figure 5:**
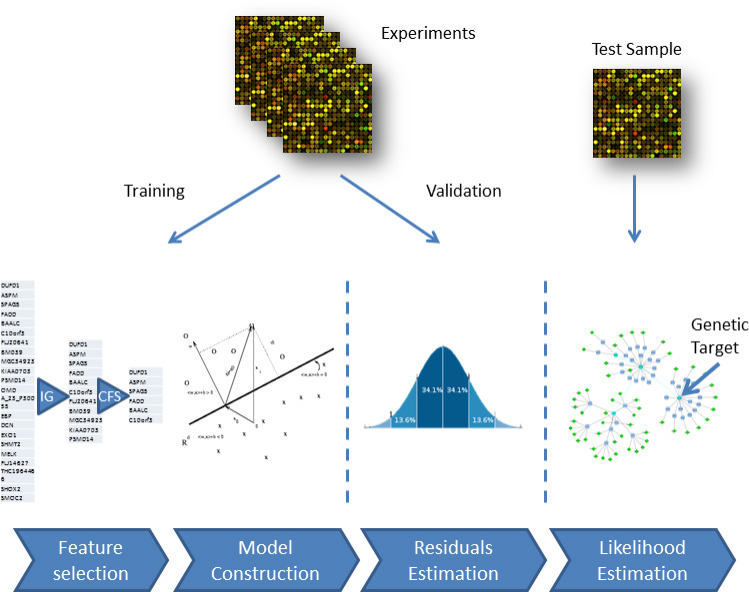
**The MoA identification workflow**.

As depicted in Figure 5, the dataset is divided in training and validation samples. For each gene a sub list of connected genes is selected using a computationally efficient algorithm. The list of genes is used to build a quantitative model of expression. This model is applied to the unseen validation samples, obtaining a predicted value for each sample. The residual difference of the real values from the predicted ones is used to compute a residual distribution describing the accuracy of the model. This distribution is used to compute the likelihood that the residual computed for the test sample is due to model inaccuracy or to a network perturbation on the given gene (details in the following sections). The algorithm has been implemented in the R statistical software and some components have been implemented in Java.

## Datasets

The whole-genome yeast expression data sets is a compendium of 300 profiles of gene deletions, titratable promoter insertions and drug compound treatments from Hughes et al. [5] and a second set of 215 titratable promoter insertions in essential genes from Mnaimneh et al. [18].

The Affymetrix yeast S. cerevisiae compendium included 904 Affymetrix Yeast Genome S98 high-density oligonucleotide arrays and 9335 probes and is available for browsing and download as yg_s98_v3_Build_2 at the Many Microbe Microarrays Database (M3D) (http://m3d.bu.edu) [19].

Simulated datasets [20] have been generated such that the network topology is produced by selecting sub networks from regulatory networks reconstructed for E. Coli and described by Ma et al. [21]. Randomly selected nodes are chosen as initial seeds. Subsequent nodes are added in an iterative process. Only randomly selected nodes that have at least one connection to the current graph are retained. The seeds are chosen and the network grows until the desired network dimension is met.

Interaction kinetics are modeled by equations based on Michaelis-Menten and Hill kinetics. These equations are used to model gene regulation in steady-state conditions: this allows on one hand to reduce the computational complexity that would have been reached with ODE models and on the other hand this kind of data is more suited for the MoA identification algorithms described so far, which require steady-state expression profiles. Biological noise is modeled by a function based on a lognormal distribution superposed on the kinetic equations. Kinetic parameters are randomly chosen from a discrete set of parameters that give place to behaviors observed in real organisms. External conditions are modeled by choosing a gene set without regulatory inputs and setting their expression level to a different value for each experiment. The expression levels of the genes in the network are subsequently calculated, as specified by their transition functions, starting from the input genes. Each connected sub network is simulated separately and the results are merged to generate datasets with a number of genes greater than the original E. Coli network. Lognormal experimental noise is added afterwards.

## Attribute selection

So far, several methods have been applied to address the network topology reconstruction issue [22]. Our attribute selection was performed in two steps. First the attributes were discretized using equal frequency bins, with a fixed number of 10 bins. After that the information gain (IG) [23] for each attribute *a *was computed as given in the following equation, where *T *is the set of samples, *H *the information entropy, *v *is one of the discretized values of the attribute/gene *a*, *x *is an instance/sample.

$$IG\left(T,a\right)=H\left(T\right)-\underset{v\in vals\left(a\right)}{\sum }\frac{\left|\left\{x\in T|x_{a}=v\right\}\right|}{\left|\left\{x\in T\right\}\right|}\cdot H\left(\left\{x\in T|x_{a}=v\right\}\right)$$

The attributes were sorted according to their information gain and only the more informative *n *attributes were retained. In the second step a correlation based feature selection [24,25] gave the list of regressors for the gene expression model. The metric for a given subset of features is given by the following equation, where *M_S _*is the heuristic "merit" of a feature subset *S *containing *k *features, $\bar{r_{cf}}$ is the mean feature-class correlation (*f *∈ *S *), and $\bar{r_{ff}}$ is the average feature-feature inter-correlation.

$$M_{s}=\frac{k\bar{r_{cf}}}{\sqrt{k+k\left(k-1\right)\bar{r_{ff}}}}$$

The search progresses forward through the search space adding single features. The search was terminated if five consecutive fully expanded subsets showed no improvement over the current best subset.

## SVM regression

The support vector machine for regression has been used to build the regression model. The parameters could be learned using an improvement of the SMO Algorithm for SVM Regression, developed by Keerthi et al. [26] The complexity parameter has been set to 0.8, the data has been normalized, the tolerance parameter has been set to 0.001, the epsilon to 1.0*E *- 12 and the kernel to polynomial of the first order.

## Filtering and target identification

The residual estimation has been performed via a bootstrap procedure. *N *sub samples were created and each subsample has been 60/40 splitted into training and test set. The models for each gene were created on the training set and evaluated on the test set. The residuals were computed as difference of the predicted and real values. The normal density function for each gene of the treated sample was computed, with mean and standard deviation computed from the residuals. The genes having a probability of belonging to the residual distribution below a given threshold were returned to the user. If the list contained more than 100 genes only the first 100 genes were returned. The threshold was adapted to the number of genes in order to control the rate of false positives.

The following pseudo-code shows how the p-values for each gene of the perturbed sample are computed from the residuals. The list of perturbed genes is obtained applying a threshold on the p-values.

for (i in 1:num_genes){

   [train, test] = sample(data)

   attributes[i] = attribute_selection(i, train)

   model[i] = estimate_model(attributes[i], train)

   predicted[i] = apply_model(model[i], test)

   observed[i] = test[i]

   residuals[i] = observed[i] - predicted[i]

   quantile = perturbed_sample[i]

   p_value[i] = pnorm (quantile,

      mean(residuals[i]),

      sd(residuals[i],

      lower. tail = quantile<0

     )

}

## Pathway analysis

The list of genes filtered by our algorithm was subsequently used for pathway analysis. Hypergeometric p-values for over or under-representation of each GO term in the specified ontology among the GO annotations for the interesting genes were computed. The computations were done conditionally based on the structure of the GO graph. For this analysis was used the *hyperGTest *function from the Bioconductor *GOStats *package [27]. The p-value cutoff was set to 0.01.

## Competing interests

The authors declare that they have no competing interests.

## Authors' contributions

VB and PP equally contributed to this work.

## Declarations

This work was partially supported by the grant of the Programma Operativo Nazionale "Ricerca e Competitività" 2007-2013 (PON "R&C") - Smart health SMART HEALTH CLUSTER OSDH - SMART FSE - STAYWELL.

This article has been published as part of *BMC Bioinformatics *Volume 14 Supplement 8, 2013: Proceedings of the 2012 International Conference on Intelligent Computing (ICIC 2012). The full contents of the supplement are available online at http://www.biomedcentral.com/bmcbioinformatics/supplements/14/S8.
